# Needle tract seeding of a sclerosing epithelioid fibrosarcoma in a biopsy tract: a case report

**DOI:** 10.1186/s12891-023-06553-0

**Published:** 2023-06-03

**Authors:** Masafumi Kawai, Shinji Miwa, Norio Yamamoto, Katsuhiro Hayashi, Akihiko Takeuchi, Kentaro Igarashi, Yuta Taniguchi, Yoshihiro Araki, Hirotaka Yonezawa, Takayuki Nojima, Hiroyuki Tsuchiya

**Affiliations:** 1 Department of Orthopedic Surgery, Graduate School of Medical Science, Kanazawa University, 13-1 Takara-machi, Kanazawa, 920-8640 Japan; 2 Department of Pathology, Graduate School of Medical Science, Kanazawa University, Kanazawa, Japan

**Keywords:** Case report, Sclerosing epithelioid fibrosarcoma, Needle tract seeding, Core needle biopsy

## Abstract

**Background:**

A sclerosing epithelioid fibrosarcoma (SEF) is an uncommon tumor of the deep soft tissue. An SEF has been described as a low-grade tumor with high local recurrence and metastatic rates. Generally, in bone and soft tissue tumors, a resection of the biopsy route is recommended; however, there is limited evidence with respect to the dissemination of the tumor tissue during a needle biopsy.

**Case presentation:**

A mass in the right pelvic cavity, with no symptoms, was observed in a 45-year-old woman during a gynecological examination. Computed tomography (CT) revealed a multilocular mass with calcification in the pelvic cavity. The magnetic resonance imaging (MRI) showed an iso-signal intensity on T1 weighted images and hypo- and iso-signal intensity on T2 weighted images. The CT-guided core needle biopsy was performed using a dorsal approach, and the biopsy diagnosis was a low-grade spindle cell tumor. The tumor was excised using an anterior approach. The tumor tissue comprised spindle cells and epithelioid cells with irregular nuclei, and the immunohistological analysis was positive for vimentin and epithelial membrane antigen, which was consistent with a diagnosis of sclerosing epithelioid fibrosarcoma. Five years after the surgery, the MRI showed a tumor recurrence in the subcutaneous tissue of the right buttock, which was consistent with the needle biopsy tract. The patient underwent a tumor excision, and the resected tumor was similar to the primary tumor.

**Conclusions:**

The recurrent tumor was excised with a surgical margin, and the tumor specimen had the histological features of a sclerosing epithelioid fibrosarcoma. It was difficult to investigate the association of the core needle biopsy with the tumor recurrence because the approach of the biopsy tract is usually same as that used in a tumor excision. However, the present case indicated the tumor may recur in the biopsy tract of a soft tissue sarcoma. Surgeons should be aware of the possibility of disseminating tumor tissues in a needle biopsy.

## Background

A sclerosing epithelioid fibrosarcoma (SEF) is a rare type of tumor characterized by the proliferation of round or oval cells arranged in cords or nests with a collagen background. An SEF was described originally in 1995 by Meis-Kindblom as an uncommon tumor in the deep soft tissues [1]. This tumor occurs on the limbs and trunk and over a wide age range, from 14 to 87 years (median age, 47 years) in both sexes [2]. While an SEF has typically low-grade histological features, it has clinically aggressive features with high local recurrences and rates of metastases, causing death in 57% of patients [3].

Although the open biopsy tract is resected commonly during a tumor resection, the need for a biopsy tract resection remains controversial. Ruiz et al. reported that an incomplete biopsy tract resection caused a higher risk of local recurrences in a study of 180 patients (15 were open and 97 were percutaneous) [4]. On the contrary, it was reported that the use of percutaneous needle biopsies did not increase any risk of local recurrences of sarcomas [5] and that the use of core needle biopsy (CNB) was not associated with the risk of local recurrences of soft tissue sarcomas in the extremities [6].

The use of needle tract seeding (NTS) from percutaneous needle biopsies has been reported in breast, liver, thyroid, renal, lung, and pancreas tumors; however, it has been difficult to ascertain whether the recurrences were due to NTS or incomplete surgical resections [7]. In this case report, we present a case with a local recurrence of a sclerosing epithelioid fibrosarcoma, and the different approaches between needle biopsy and tumor resection indicated the recurrence was caused by needle biopsy tract seeding.

## Case presentation

**Fig. 1:**
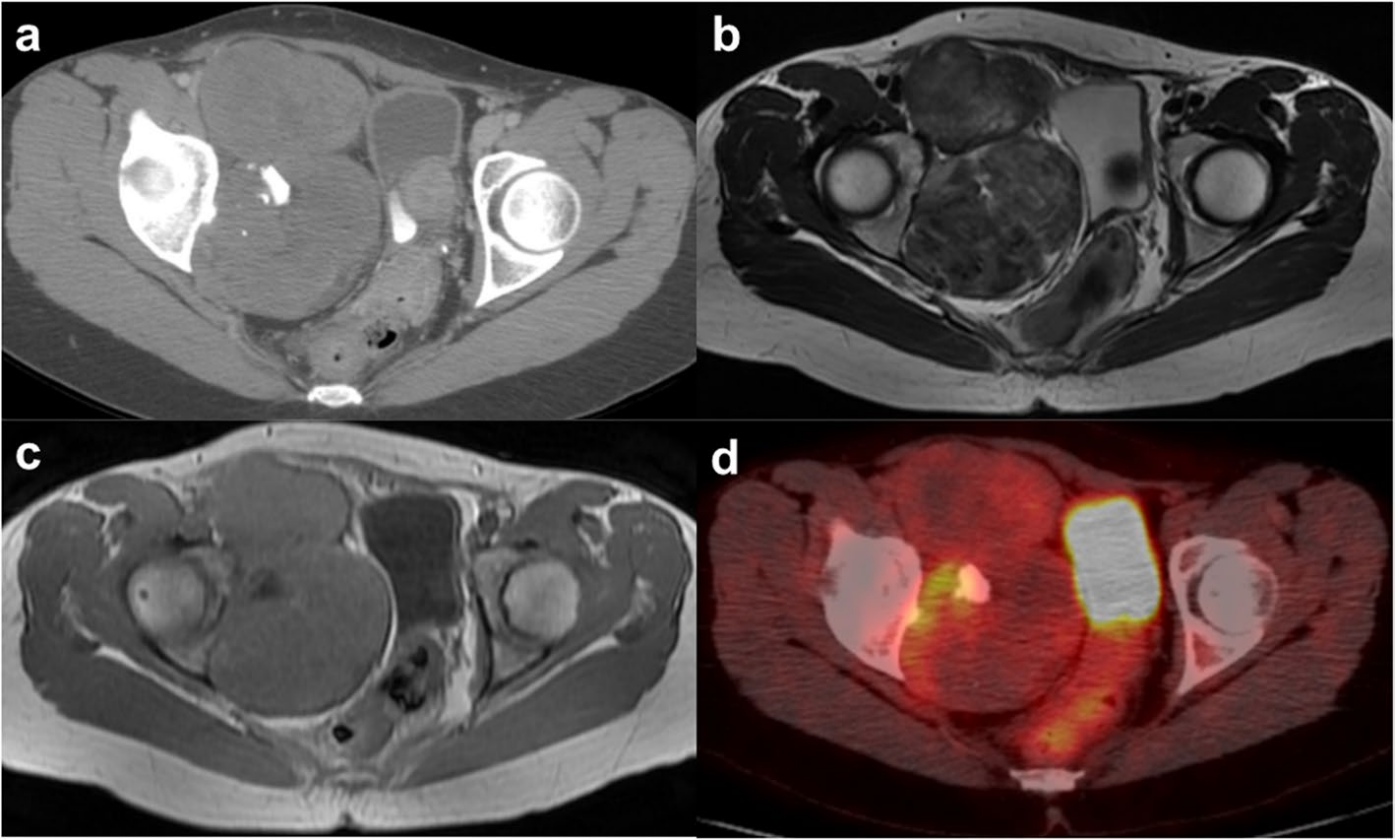
Radiologic image findings before the first surgery. Computed tomography (CT) showing a multilocular mass with calcification in the pelvic cavity (**a**). Magnetic resonance imaging (MRI) demonstrating iso-signal intensity on T1 weighted images (**b**) and hypo- and iso-signal intensity on T2 weighted images (**c**). ^18^Fluorine-labeled fluorodeoxyglucose–positron emission tomography showing an increased uptake of ^18^ F-fluorodeoxyglucose in the calcified part (SUV = 6.5) while no increased uptake is seen in other lesions (**d**)

**Fig. 2:**
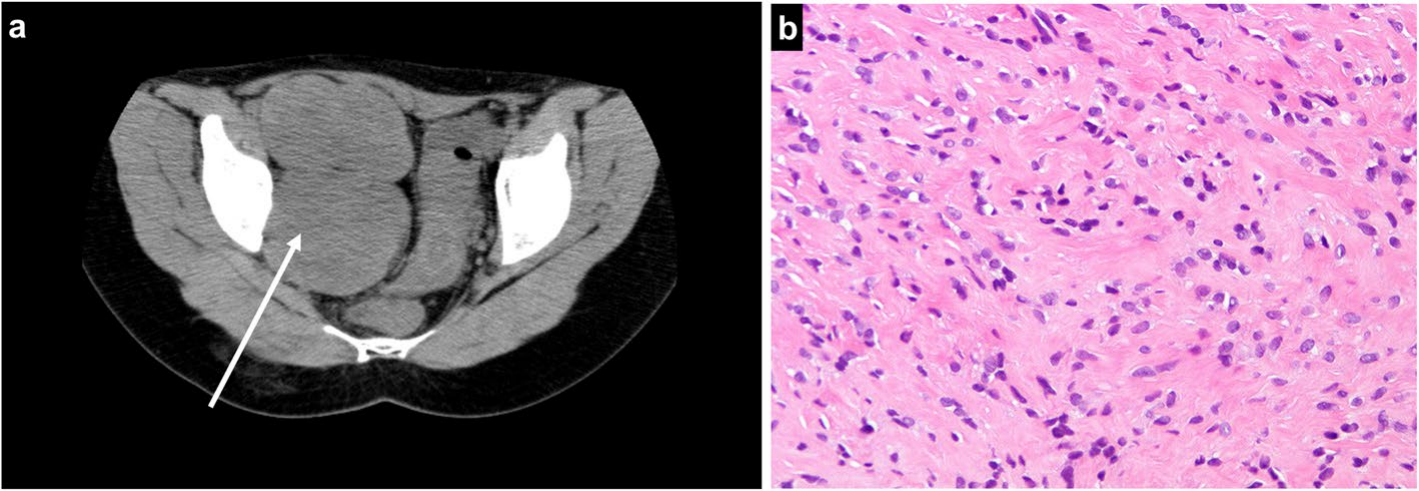
CT at CT-guided core needle biopsy (CNB) through posterior approach (arrow) (**a**). Histology at CT-guided CNB. Hematoxylin and eosin staining showing a proliferation of the spindle cells with no atypical cells and a low cell density (**b**)

A mass in the right pelvic cavity, with no symptoms, was observed in a 45-year-old woman during a gynecological examination. The patient was referred to our hospital due to tumor growth, despite 6-month hormone therapy. On physical examination, a palpable abdominal mass, which was hard and immobile, was observed. Computed tomography (CT) revealed a multilocular mass with calcification in the pelvic cavity (Fig. 1a). Magnetic resonance imaging (MRI) showed an iso-signal intensity on T1 weighted images and hypo- and iso-signal intensity on T2 weighted images (Fig. 1b, c). Positron emission tomography (PET) showed an increased uptake of ^18^ F-fluorodeoxyglucose in the calcified part with no increased uptake in the other lesions (Fig. 1d). It was necessary to collect tissue from a calcified lesion inside the tumor, which was found to be accumulated on PET-CT. Because the ventral approach had risks of vascular and organ damages and dissemination of tumor tissues in the intraperitoneal space, the patient underwent CT-guided CNB using the dorsal approach with an 18-gauge needle, (Fig. 2a). The CNB procedure was repeated twice and no subcutaneous hematoma was observed after the needle biopsy. Spindle cells with no atypical cells and with a low cell density were observed and a spindle cell tumor of benign or low-grade characteristic was suspected (Fig. 2b).

**Fig. 3:**
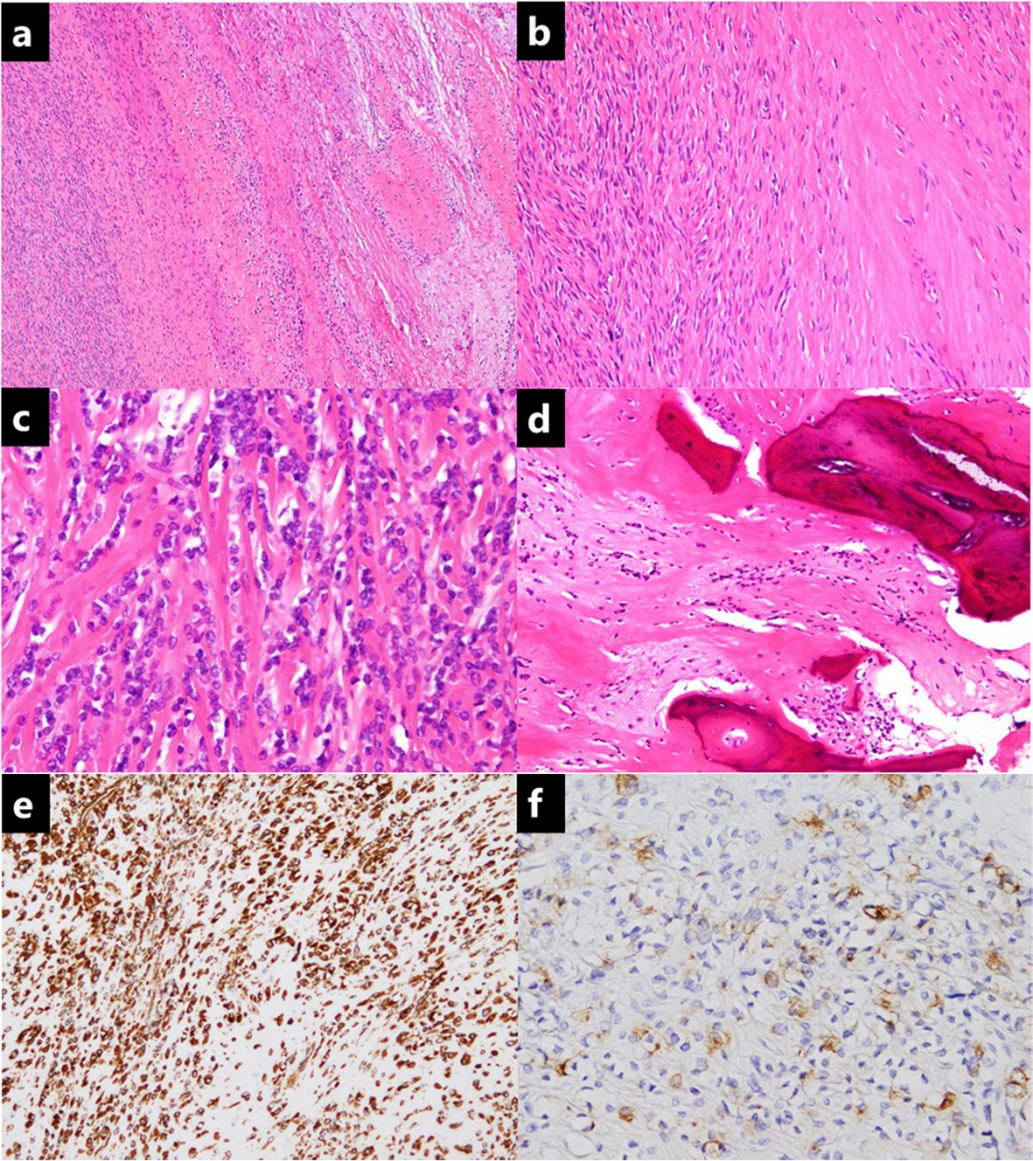
Histology at surgical treatment. Hematoxylin and eosin staining showing a proliferation of spindle-shaped cells with a high cell density in the hyalinized stroma, collagen fiber hyperplasia with hyalinization, and mucinous degeneration (**a**, **b**, **c**). Hyalinized stroma with myxoid change and metaplastic bone is observed (**d**). In immunostaining, vimentin (**e**) and epithelial membrane antigen (focal and weak) (**f**) are positive

The patient underwent marginal excision through the anterior approach. The tumor was located close to the abdominal wall and wrapped by a capsule, and there was no adhesion to the surrounding tissue. The size of the resected tumor specimen was 13 × 10 × 12 cm. Pathologically, the tumor was composed of short spindle cells with relatively uniform epithelioid cells arranged in cords and nets. The nuclei showed occasional irregularity and enlargement (Fig. 3a, b, c, d). The immunohistological analysis was positive for vimentin and epithelial membrane antigen (focal and weak) and negative for MUC4, S-100, desmin, CD34, and β-catenin (Fig. 3e, f). Based on these histopathological findings, the diagnosis of the tumor was that of a sclerosing epithelioid fibrosarcoma.

Three years after the surgery, an enhanced MRI revealed a recurrent tumor in the right obturator muscle and the patient underwent heavy ion therapy.

**Fig. 4:**
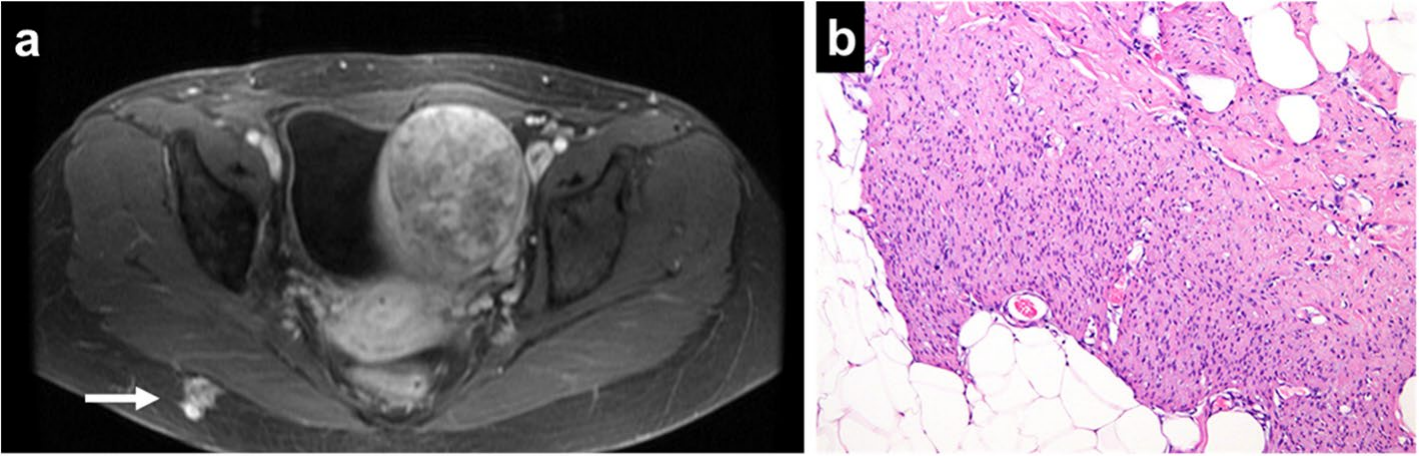
MRI showing the recurrent tumor in the subcutaneous tissue of the left buttock. The tumor was distant from the approach of the surgical resection of the primary lesion and matched with the CNB tract (**a**). The histology of the recurrent tumor. The proliferation of the spindle-shaped cells with the hyalinized collagen fiber within the subcutaneous adipose tissue (**b**)

Five years after the surgery, the MRI showed a recurrent tumor in the subcutaneous tissue of the left buttock, measuring 1.2 cm at the greatest diameter (Fig. 4a). The site of the recurrent tumor was distant from the approach of the surgical resection of the primary lesion and was matched with the CNB tract. Therefore, it was considered that the tumor recurrence was caused by NTS. The patient underwent a wide excision of the recurrent tumor. The resected specimens had similar features to the specimens of the primary tumor (Fig. 4b).

Approximately 2 years after the second surgery, the patient was alive and experienced no recurrence.

## Discussion and conclusions

An SEF was first described in 1995 by Meis-Kindblom [1]. The lower extremities/limb gridle, trunk, upper extremities, and head and neck were frequent tumor sites [2, 8]. An SEF occurs commonly in the deep soft tissue and is associated with the adjacent fascia or periosteum; however, there have been few reports of tumors arising from the bone [9]. In our case, the tumor was wrapped in a capsule and although it could be detached easily from the surrounding tissues, partial continuity with the pelvic bone and the possibility of tumors arising in the bone could not be ruled out. The pathological examination did not reveal whether it originated from bone, periosteum, or fascia. An SEF has been described mainly as a low-grade sarcoma, although high local recurrence rates (36 to 100%) and distant metastatic rates (43 to 90.9%) were reported [1–3, 10, 11]. In a retrospective literature review, the most common site of a distant metastasis was the lung, followed by the bone, pleura, chest wall, liver, breast, and brain [2, 11]. Antonescu et al. have reported a case of soft-tissue metastasis [3]; however, as in our case, there are few cases of metastasis to the subcutaneous tissue. In a report on fine needle biopsy (FNB) for thyroid cancer, the interval between FNB and seeding ranged between 2 months and 11 years. A previous report on hepatocellular carcinoma also showed that the time from needle biopsy to detection of implantation ranged from 2.3 to 85.8 (median, 26.8) months [12]. Thus, there is a time range from needle biopsy to tumor recurrence in various studies. One SEF study reported that local recurrence occurred at a median of 26 months (range: 3–178 months), metastasis occurred at a median of 10.5 months (range: 0–192 months), and the tumors were relatively slow-growing [13]. Therefore, in our case, although it has been 5 years since the needle biopsy, we consider this tumor to be a recurrence due to needle biopsy, given the nature of the tumor and its presence on the needle biopsy route.

Histologically, an SEF has been characterized by the presence of small, acidophilic, epithelioid-like cells arranged in a cord or focal pattern within a hyalinizing collagenous matrix. Cellular atypia is not pronounced and nuclear fission is poor. Ossification may also be observed in the hyalinizing stroma [1, 3, 9]. Occasionally, parts similar to a low grade fibromyxoid sarcoma (LGFMS) have been found, and cases with a mixed histology of LGFMS and SEF, or cases of LGFMS with SEF-like images at recurrence and their associations have been reported [3]. MUC4, which is highly specific for LGFMS, has been expressed in 78% of cases and is important in the diagnosis of an SEF. A pure SEF has showed a MUC4 expression in 69% of cases, while in hybrid LGFMS-SEF tumors, 100% of the cases showed a strong diffuse MUC4 expression [14]. Therefore, in this case, it was considered that the MUC4 was negative, since it is the pure SEF that presents the typical histology of an SEF. Discrimination between an SEF and a LGFMS is important because an SEF is considered to be more malignant than an LGFMS [14].

NTS is known in a variety of cancers, including breast, thyroid, liver, pancreas, and kidney cancer, but has been reported as a rare complication. However, the incidence of NTS after the percutaneous biopsy has been very low from 0.003 to 0.007% for pancreatic tumors and 0.4 to 5.1% for liver tumors [7]. In another study on head and neck neoplasms, excluding that of the thyroid and parathyroid, the incidence of NTS after a percutaneous biopsy was estimated at 0.00012–0.0011% [15]. In contrast, very few reports were available on NTS of the bone and soft tissue sarcoma. Davis et al. reported, for the first time, the recurrence of osteosarcoma in the needle biopsy tract [16]. In a retrospective study on the incidence of NTS, Houbt et al. reported that five patients of 255 patients (2%) developed NTS in the retroperitoneal sarcoma such as a leiomyosarcoma and liposarcoma [17]. This is the first report of NTS of SEF. Although SEF has been reported to be a low-grade sarcoma, high local recurrence and distant metastasis rates have been reported. The aggressiveness of this tumor can be associated with NTS in this case. In previous reports, NTS has been reported in aggressive tumors, including hepatocellular, pancreatic, lung, and breast cancer [18]. Tumor aggressiveness may be associated with the risk of NTS, and NTS in papillary thyroid cancer has been reported to be considerably less common than in liver and pancreatic cancer, which are generally more aggressive [19]. It has also been reported that NTS may occur more frequently in soft tissue tumors compared to bone tumors. The high cell number and low matrix content characteristic of soft-tissue sarcomas may be related to their greater cell spread compared to bone tumors [20]. In this case, NTS occurred in the subcutaneous tissue of the buttock, but there are no studies on the site of NTS. To our knowledge, there have been no reports of NTS being more likely to occur in subcutaneous adipose tissue or of an association between hematomas and NTS. Although one case of a hematoma following CNB and subsequent NTS has been reported, there was no evidence of an association between hematomas and NTS [21].

Although it is not possible to quantify definite risk factors for NTS because of very few reported cases; needle diameter (gauge), type of needle (cutting/non-cutting), number of biopsies (passes), and characteristics of neoplastic cells have all been considered as possible risk factors for NTS [22, 23]. An increase in the number of needle punctures to two may increase NTS and needle exchange and cleaning is recommended [23, 24]. The following biopsy principles have also been advocated for the prevention of NTS: the biopsy site should be in close proximity to the primary site, disruption of the fascial surface should be minimized, surrounding vital structures should be avoided and every effort should be made to avoid contamination of free spaces such as the abdominal cavity [17]. As our case also involved two punctures, NTS could have been avoided by collecting multiple cores with a single puncture using the co-axial biopsy technique [17, 25]. A CNB must be performed along with the standard surgical incision at the site and a tumor excision with a surgical margin including the biopsy tract is recommended in cases of sarcoma. However, tumor excisions with biopsy tracts are sometimes difficult for various reasons. In our case, the biopsy tract was not excised because the CNB was performed using the dorsal approach to avoid any vascular and organ injury. Therefore, we had to select an excision route that was completely different from the biopsy route, due to the structure of the pelvis. Houbt et al. found that the risk of NTS in a retroperitoneal sarcoma was slightly higher than that observed in previous studies on cases with extremity sarcomas because the biopsy tract is excised rarely in retroperitoneal sarcomas [17]. Furthermore, other reasons included the proximity of the biopsy route to the nerves and blood vessels, and with respect to cosmetic issues in the head and neck tumors. In contrast, there were some reports that indicated that a needle biopsy tract excision was not associated with the recurrence of the tumor. In a study on 59 cases of high-grade soft tissue sarcoma without a resection of the CNB tract, there were five local recurrences (9%) and 15 metastases (25%). They found no increase in the local recurrence and metastases rates compared with the findings of previously published studies [10]. Saghieh et al. reported no local recurrences in 10 cases of osteosarcoma without a resection of the CNB tract during an average follow-up period of 4 years. They suggested that the incidence of NTS in the bone sarcoma without an excision of the CNB tract may not be high enough to warrant an excision of the entire tract [26]. In a study of 116 cases of extremity soft tissue sarcomas, a comparison of 36 patients who underwent a CNB tract resection and 36 patients who did not, indicated that there was no significant difference in the recurrence rate [6]. Thus, the necessity of a needle tract resection has been controversial. Furthermore, one of the reasons that a needle tract resection remains controversial is that it cannot be denied residual tumor status after a tumor excision or soft tissue metastasis. In our case, we denied the residual tumor status after the tumor excision due to the use of different approaches to the CNB and the excision. A recurrent tumor was observed along the biopsy route and the site of recurrence was distant from the location of the primary tumor and surgical route. Moreover, the course of development and histopathology showed a strong indication of NTS.

In conclusion, we observed a valuable case of SEF that could deny a residual tumor status after a surgical excision and a diagnosis NTS. NTS is infrequent and it remains controversial whether a biopsy tract excision is necessary or not; however, if the biopsy tract is not excised as in this case, surgeons should be alert to the possibility of a recurrence in the needle biopsy tract.

## Data Availability

To protect the privacy and to respect confidentiality, no raw data have been made available in any public repository. The datasets used and/or analyzed during the current study available from the corresponding author on reasonable request.

## Abbreviations

SEF: Sclerosing epithelioid fibrosarcoma
CT: Computed tomography
CNB: core needle biopsy
NTS: Needle tract seeding
MRI: Magnetic resonance imaging
LGFMS: low grade fibromyxoid sarcoma
